# Sequential miRNA-mRNA profiling of regulatory pathways during in vitro maturation of canine oocytes

**DOI:** 10.1590/1984-3143-AR2026-0003

**Published:** 2026-08-31

**Authors:** Monica De los Reyes, Phillip Dettleff, Jaime Palomino, Oscar Alejandro Peralta, Victor Hugo Parraguez, Belen Orellana, José Miguel Avendaño

**Affiliations:** 1 Laboratorio de Reproducción Animal, Facultad de Ciencias Veterinarias, Universidad de Chile, Santiago, Chile; 2 Escuela de Medicina Veterinaria, Facultad de Ciencias de la Salud, Universidad Bernardo O’Higgins, Santiago, Chile; 3 Escuela de Medicina Veterinaria, Facultad de Agronomía e Ingeniería Forestal, Pontificia Universidad Católica de Chile, Santiago, Chile; 4 Laboratorio de Fisiología Animal, Facultad de Ciencias Veterinarias, Universidad de Chile, Santiago, Chile

**Keywords:** culture, dog, gametes, transcripts, sequencing

## Abstract

The acquisition of meiotic and developmental competence in oocytes is a highly regulated process that includes post-transcriptional mechanisms mediated by microRNAs (miRNAs). To characterize these changes, we compared mRNA and miRNA expression profiles between non-matured, and oocytes submitted to IVM in canines using RNA sequencing. The oocytes were obtained from antral follicles dissected from canine ovaries. Total RNA was isolated from pooled oocytes from each condition and subjected to parallel mRNA and miRNA sequencing, followed by differential expression and pathway enrichment analyses. Our findings reveal transcriptional change after in vitro culture, showing significant differential expression (FDR < 0.05, |log_2_FC| > 1). Non-matured oocytes displayed an expression pattern associated with transcriptional preparation and structural organization, while IVM oocytes showed expression patterns consistent with cytoplasmic remodeling and metabolic activation. The miRNA profile changed with maturation, accompanied by a selective increase in miRNAs associated with post-transcriptional control of the cell cycle and structural pathways. Enrichment analysis revealed significant changes in signaling networks associated with cell adhesion, cytoskeletal organization, and communication. Components of the Hippo signaling pathway displayed altered transcript abundance following IVM, suggesting that structural and regulatory cues present within the oviduct may not be fully reproduced in culture. Our findings provide new insights into the molecular constraints of canine IVM that may serve as biomarkers for improving oocyte quality and reproductive technology outcomes in dogs.

## Introduction

Oocyte maturation is a critical step for successful fertilization and embryo development in mammals, requiring both nuclear and cytoplasmic changes that are tightly regulated at the molecular level (He et al., 2021). Much of this regulation is mediated by microRNAs (miRNAs), small non-coding RNAs that post-transcriptionally regulate gene expression by binding to complementary sequences in target mRNAs, leading to their degradation or translational inhibition (Bartel, 2004; Mishima and Tomari, 2016)

In domestic dogs, oocyte development has a unique reproductive pattern, as ovulated oocytes are released at the germinal vesicle (GV) stage and complete meiotic maturation to second metaphase (MII), only after ovulation within the oviduct (Reynaud et al., 2005; Saint-Dizier et al., 2020). This species-specific delay in nuclear maturation significantly complicates the development of efficient in vitro maturation (IVM) protocols, which consistently yield oocytes with poor developmental competence compared to those matured in vivo (Nagashima and Songsasen, 2021; De los Reyes et al, 2023). The biological reasons for this inefficiency remain unresolved.

Recent evidence suggests that miRNA expression is dynamically regulated during oocyte maturation and that specific miRNA-mRNA networks are critical for the successful progression of meiosis, modulation of apoptosis, and maintenance of oocyte quality (Nazou et al., 2024) Moreover, the differential expression of miRNAs has been associated with oocyte competence in bovine (Abd El Naby et al., 2013) and human models (Xu et al., 2015), suggesting their potential as biomarkers or modulators of development. However, in the canine species, the miRNA regulatory condition and its interaction with mRNA targets during oocyte maturation have not been systematically explored. Recent sequency RNA studies in our laboratory, analyzing the transcriptomic profiles of miRNAs and mRNAs in canine cumulus cells (CCs) and oocytes during in vitro maturation, revealed 643 significant miRNA-mRNA co-expression relationships, suggesting that miRNAs play pivotal roles in regulating mRNA expression during oocyte maturation (Dettleff et al., 2025); however, the post-transcriptional regulation changes during IVM of canine oocytes compared to non-matured oocytes have not been reported. To address this, we hypothesized that IVM of canine oocytes is associated with different miRNA-mRNA regulatory networks compared to non-matured oocytes, and that disruption of these post-transcriptional interactions underlies the limited developmental competence observed under IVM conditions. To test this hypothesis, we performed a sequential miRNA-mRNA expression analysis comparing non-matured and in vitro matured canine oocytes. By integrating differential expression profiles with pathway enrichment analyses, this study aimed to identify key molecular signatures and regulatory interactions that may explain the limited success of current IVM protocols in dogs, with special attention to miRNAs and mRNAs with significant expression changes and their potential roles in signaling pathways known to influence oocyte development.

## Methods

### Animal and sample collection

Oocytes were obtained from the ovaries of adults (1-4 years old), mixed-breed, clinically healthy bitches (n=108) at different estrous stages, after routine ovariohysterectomy at the local Veterinary centers near our laboratory. Ovarian samples were obtained in accordance with the guidelines of the Animal Care Committee, University of Chile (CICUA: 25930-VET-UCH). All samples used in this study were collected with the informed consent of the dog owners.

### Ovarian processing and oocyte retrieval

Within 30 to 60 minutes after surgery, the ovaries were transported to the laboratory in physiological sterile saline solution (NaCl 0.9%) supplemented with 100 IU/mL penicillin and 20 µg/mL streptomycin (Merck, Darmstadt, Germany) at 4 °C.

At the laboratory, each ovary was washed in PBS (#P2272, Sigma-Aldrich. St. Louis, MO, USA), and only ovaries without visible alterations were selected. The antral follicles were dissected under a stereomicroscope (Meiji Techno SKT, Tokyo, Japan). The cumulus-oocytes complexes (COCs) were released from 0.5 mm to 4.9 mm antral follicles, assessed by a grid in the eyepiece of the magnifier (García et al., 2019), from late anestrus and proestrus by puncturing them with a narrow-bore Pasteur pipette. The COCs were placed in 35 mm petri dish (Falcon # 3001; Becton Dickinson, Lincoln Park, NY, USA) containing TCM-199 medium (Earle’s salt, buffered with 25mM Hepes. Sigma-Aldrich. St. Louis, MO, USA), supplemented with 10% of FCS (Fetal Calf Serum. Sigma- Aldrich) and 100 μg/mL of streptomycin (Sigma-Aldrich) and examined under a dissecting microscope (Meiji Techno SKT, Tokyo, Japan). After three washes in this medium, COCs were aspirated using a fine-tip Pasteur pipette and washed with PBS-FCS solution. They were then selected according to previously described criteria (De los Reyes et al., 2023), based on dense, homogeneous, and dark cytoplasm, with more than 2 layers of cumulus cells surrounding the oocyte. COCs were grouped separately as non-matured and in vitro-matured oocytes.

Oocytes not submitted to IVM were carefully denuded of their cumulus cells by gently pipetting, thus achieving separation of the cumulus cells and oocytes. Denuded oocytes were stored in RNA later (# K0731, Thermo Scientific).

### In vitro maturation

All oocytes submitted to IVM, were cultured for 72 h in TCM-199-Hepes, Earle’s salt, (12340-030 Gibco, Thermo Fisher, Grand Island, NY, USA) supplemented with 10% fetal calf serum (FCS, #A6003), 10 IU mL^-1^ human chorionic gonadotropin (hCG #CG10), 0.25 mM pyruvate 5µL/mL, 2 μg/mL ß-estradiol (E8875–1G), 50 μg/mL progesterone (#P7556) 100 IU/mL penicillin (Sigma #K0521), and 20 μg/mL streptomycin (#S9137) (all from Sigma-Aldrich) at 38 °C and in a humidified atmosphere of 5% CO_2_. In each biological replicate, COCs were placed in 100 μL culture drops containing no more than 8 COCs, and the culture drops were covered with mineral oil (Sigma-Aldrich #M8410-1L).

After culturing, each COC was examined under an inverted microscope (Nikon, Tokyo, Japan). Only expanded COCs were used as IVM oocytes; cumulus cells were mechanically removed from the COCs by passing them through a narrow glass pipette. Denuded oocytes were then kept in RNAlater (Ambion Invitrogen, Eugene, OR, USA) and stored at -80 °C.

Oocytes from either group (non-cultured or IVM) were submitted to total RNA isolation for further analysis.

### RNA extraction, integrity evaluation, and library preparation

Three pools of around 300 IVM oocytes each, and three other pools of 300 from non-matured oocytes were subjected to RNA extraction using the GeneJet RNA Purification Kit (# K0731). The large-scale pooling strategy derived from 108 donors was designed to avoid individual differences. The concentration of total RNA in each pool was measured using a Qubit Fluorometer (Invitrogen, Eugene, OR, USA) with the Qubit RNA Assay Kit (Molecular Probes, Invitrogen) (De los Reyes et al., 2024). RNA purity was assessed by measuring the 260/280 nm absorbance ratio using an Epoch Spectrophotometer System (BioTek, Agilent Technologies, Santa Clara, CA, USA). RNA integrity was evaluated using the Fragment Analyzer system (Advanced Analytical Technologies) with the Standard Sensitivity RNA Analysis kit. Only samples with an RNA Quality Number (RQN) greater than 8 were selected for sequencing analysis (Dettleff et al., 2025). RNA samples were labeled and stored at -80 °C.

Total RNA was used for the library construction and sequencing of mRNA and miRNA Libraries were quantified with the Kappa Library Quantification kit (Roche, NJ, USA) by qPCR, and library size was determined with the Bioanalyzer, while miRNA libraries were generated with the TruSeq Small RNA Library Preparation Kit (Illumina, San Diego, CA, USA).

### Sequencing, filtering, and mapping of miRNA and mRNA

miRNA and mRNA libraries were sequenced on the HiSeq 2500 (Illumina) platform, using pair-end sequencing for mRNA (2x150 bp) and single-end sequencing for miRNA (1x50 bp) on an external sequencing platform (Macrogen), as previously described (Dettleff et al., 2025).

Raw sequencing reads were initially assessed for quality using FastQC software. Trimming was performed with Trimmomatic v0.22 to remove low-quality bases, sequencing adapters, and short reads (<15bp). Filtered mRNA reads were aligned to the *Canis lupus familiaris* reference genome (assembly UU_Cfam_GSD_1.0; GenBank accession GCF_011100685.1, NCB1) using Kallisto for transcript quantification. For miRNAreads, mapping was performed against the *Canis lupus familiaris* reference miRNA dataset from miRBase using the miRDeep2 package (Mackowiak, 2011)

### Analysis of differential expression

To determine expressed miRNAs in non-matured oocytes and IVM oocytes, the normalized expression values of miRNA from mirDeep2 were used. The differences between these two types of oocytes were determined for transcripts (miRNAs or mRNAs) with adjusted p-value, using the Benjamini-Hochberg method (Benjamini and Hochberg, 1995) of 0.05 and absolute log2 fold-change >1. Genes ID of differentially expressed mRNA were used for DAVID GO enrichment and KEGG pathway analysis (Dettleff et al., 2025).

## Results

**Figure 1:**
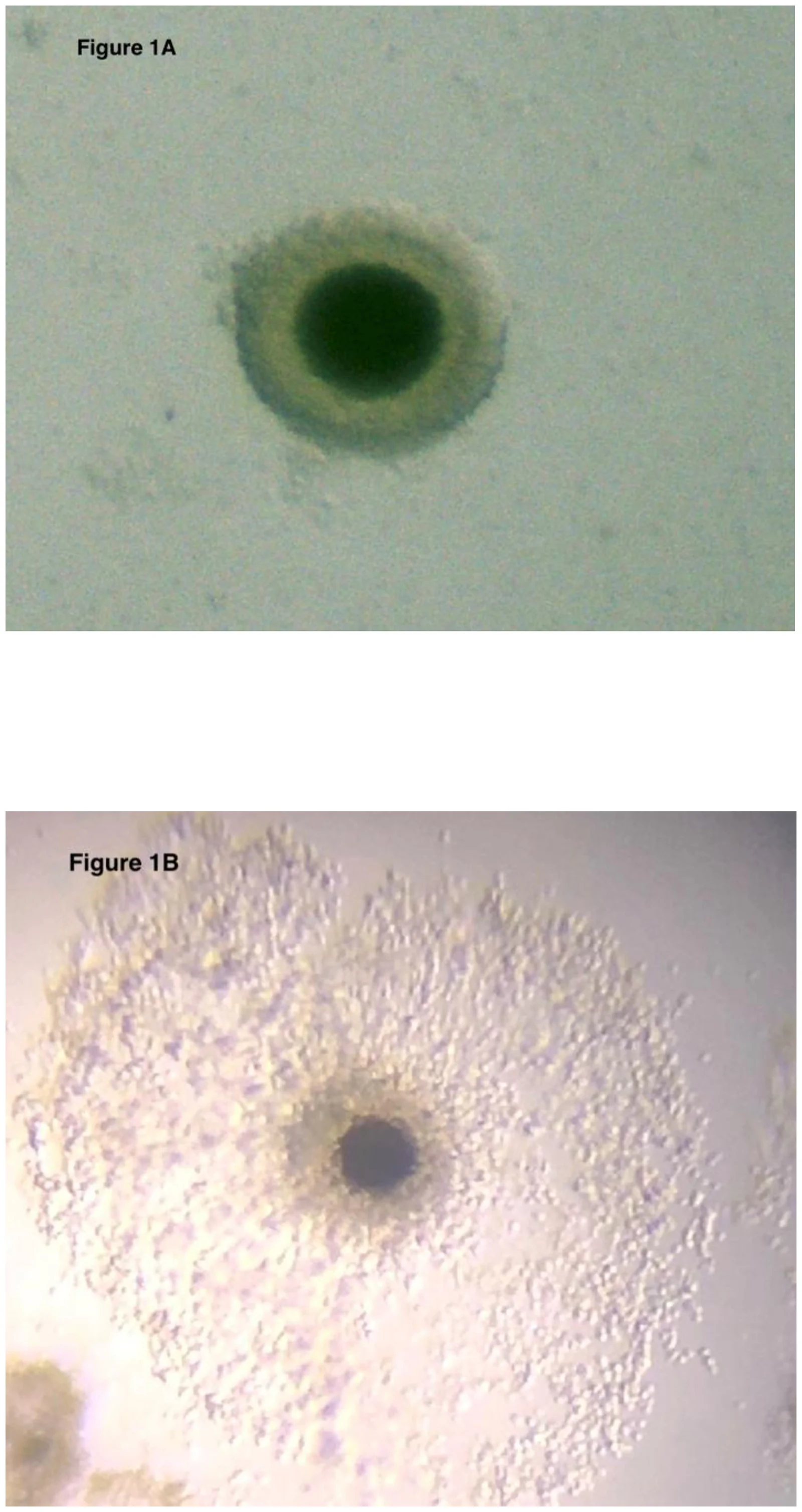
Canine cumulus–oocyte complexes (COCs) before (A) and after 72 h of in vitro maturation (B). Only those oocytes with the cumulus cells expanded were used as IVM oocytes.

Non-matured oocytes (Figure 1A) and those matured in vitro that expanded cumulus cells (Figure 1B) were processed for sequencing analysis.

The description and data analysis comparing the gene expression between non-matured and IVM canine oocytes**,** based on log2 fold change (log2FC) and adjusted *p*-values, are shown in Table S1, Supplementary Material. The majority of DEGs have adjusted *p* < 0.01, showing important statistical significance. Both negative and positive fold changes indicated that some genes were highly expressed in non-matured oocytes, and others were highly expressed in IVM oocytes. Many differentially expressed transcripts exhibited a large fold change after adjustment for multiple testing. *NAA35* and *MAZ* were the most regulated genes, indicating downregulation in the non-matured versus IVM oocytes. *KDM6B***,** which encodes a histone *H3K27* demethylase, and *ZNF236***,** *RNF128****,*** *ZNF292, SUN1*, and *PUM2*, were down regulated in non-matured oocytes. In contrast, several other genes were upregulated in non-matured oocytes compared with IVM, such as *REPS1* and *BDP1*. Additional transcripts such as *RELL2****,*** *SORT1****,*** *AGO4***,** *PTPRF***,** *KIDINS220*, and *SDC1* also exhibited positive fold changes (*p*<0.05). A massive transcriptional reprogramming occurred after culture. Other down-regulated genes in non-matured oocytes were transcription factors**,** signaling components**,** ubiquitin modifiers, and cytoskeletal regulators.

The differential expression of miRNAs between non-matured and IVM canine oocytes is presented in Table 1, using log2FC (*p* < 0.05). There was a change in the post-transcriptional regulatory condition during IVM. In total, 14 miRNAs showed differential expression (*p* < 0.05) when comparing non-matured vs IVM canine oocytes. Four miRNAs: *cfa-miR-224***,** *cfa-miR-494***,** *cfa-miR-10b*, and *cfa-let-7g*, were downregulated in non-matured oocytes relative to IVM (*p* < 0.05). The remaining 10 miRNAs were upregulated in non-matured oocytes, with fold changes ranging from ~1.04 to ~2.00. The highest positive log_2_FC was observed for *cfa-miR200c*, followed by *cfa-miR-19b* and *cfa-miR-151*, indicating miRNA expression change during oocyte maturation.

**Table 1:** Differential expression of miRNAs between non-matured And in vitro matured canine oocytes.

**miRNA**	**Immature oocyte vs IVM oocyte log2FC**	**Immature oocyte vs IVM oocyte adjPval**
CFA-MIR-224	-1.2851453	0.00023651
CFA-MIR-494	-1.2391059	0.04966748
CFA-MIR-10B	-1.1125018	0.00189679
CFA-LET-7G	-1.0425034	0.00099943
CFA-MIR-214	1.04152477	0.00608681
CFA-MIR-574	1.16631904	0.01827261
CFA-MIR-106B	1.18333649	0.04955539
CFA-MIR-451	1.26162812	8.25E-05
CFA-MIR-101	1.28852442	0.03135769
CFA-MIR-29C	1.39415084	0.04505198
CFA-MIR-202	1.46573578	8.25E-05
CFA-MIR-151	1.54216532	0.00042738
CFA-MIR-19B	1.94380428	0.00279507
CFA-MIR-200C	2.00447887	0.00099943

**Figure 2:**
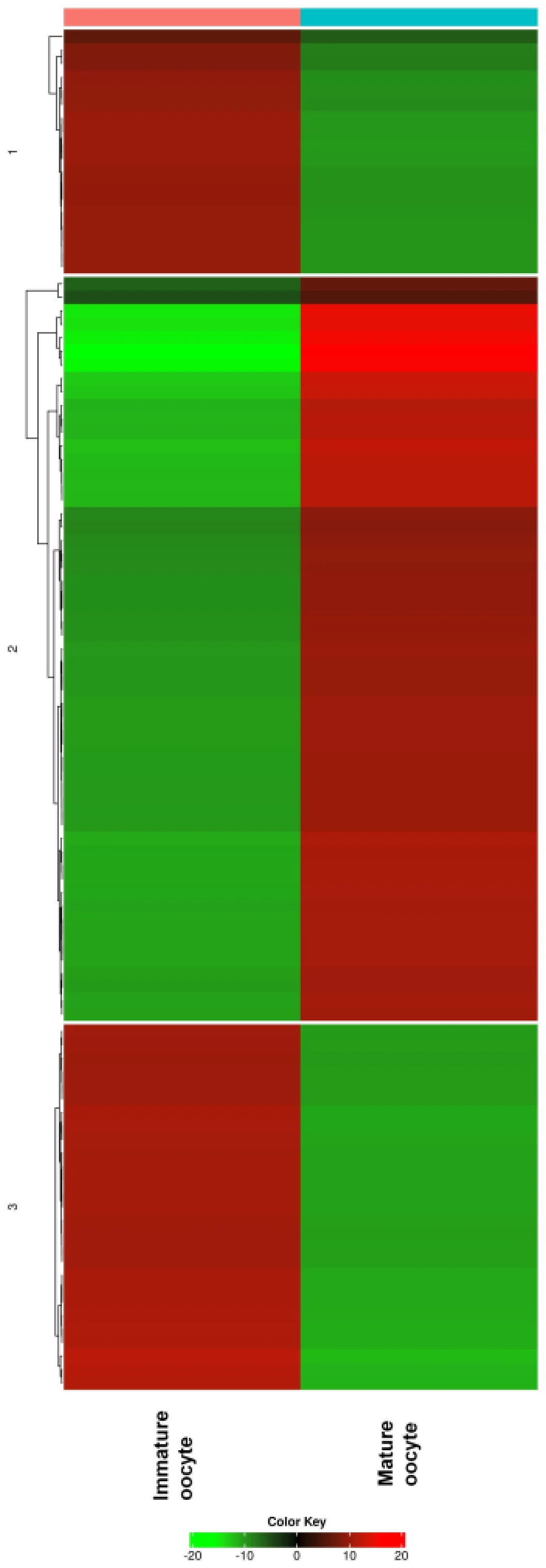
Heatmap displaying the differential expression of mRNAs between non-matured oocytes and in vitro matured oocytes.

The heatmap in Figure 2 displays the differential expression of mRNAs between non-matured oocytes and IVM oocytes. The color scale ranges from -5, indicating downregulation, to +10, meaning upregulation, suggesting transcriptional differences between non-matured and IVM oocytes. The downregulation, which was dominant in non-matured oocytes compared to upregulation in IVM samples, implicates that many genes were upregulated after IVM. In contrast, some other transcripts were highly downregulated in IVM oocytes compared to non-matured oocytes.

**Figure 3:**
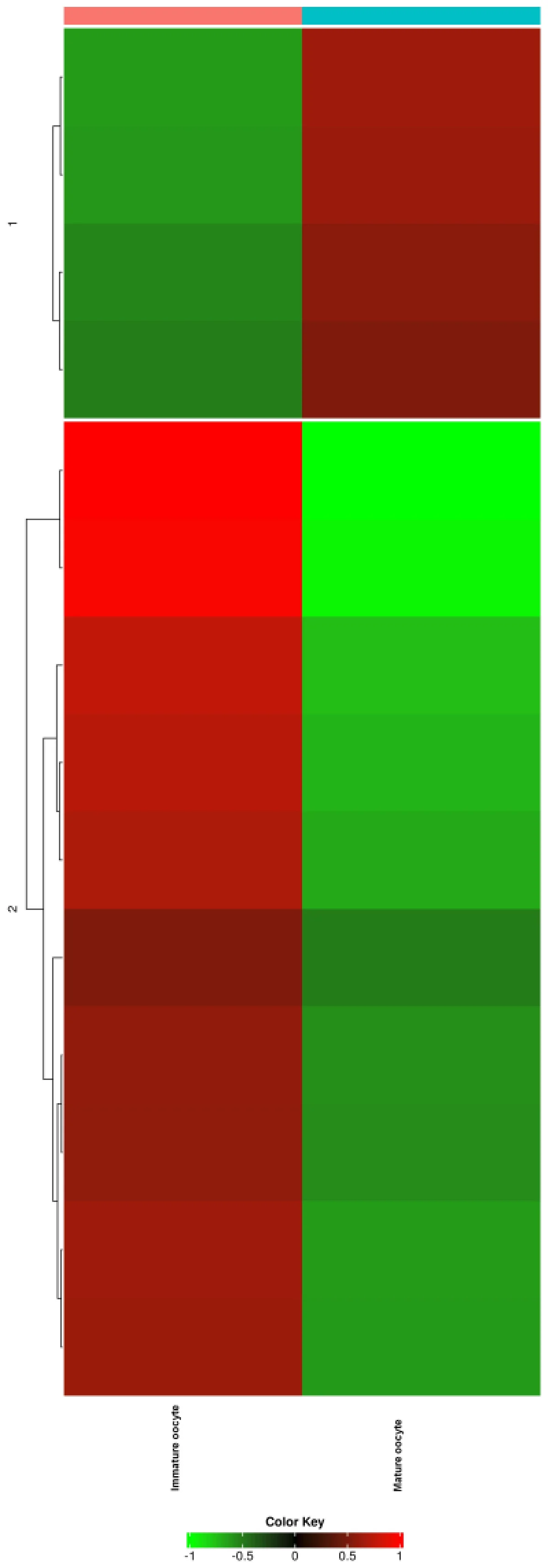
Heatmap of differential expression of miRNAs between non-matured oocytes and IVM oocytes.

The miRNA expression profiles in canine oocytes before, in the left column, and after IVM, in the right column, are shown in the heatmap of Figure 3. A non-matured oocyte is dominated by widespread downregulated expression with negative values, whereas an IVM oocyte turned to upregulated. The heatmap revealed two clusters: cluster 1, located in the top rows, displayed miRNAs with moderate activation upon IVM, possibly involved in general readiness for meiosis; and cluster 2, situated in the lower rows, exhibited pronounced expression changes, potentially including regulators of cytoplasmic and nuclear maturation. The change from green or down-regulated to red or up-regulated across these clusters indicated reprogramming of miRNA-mediated gene regulation during oocyte maturation.

**Figure 4:**
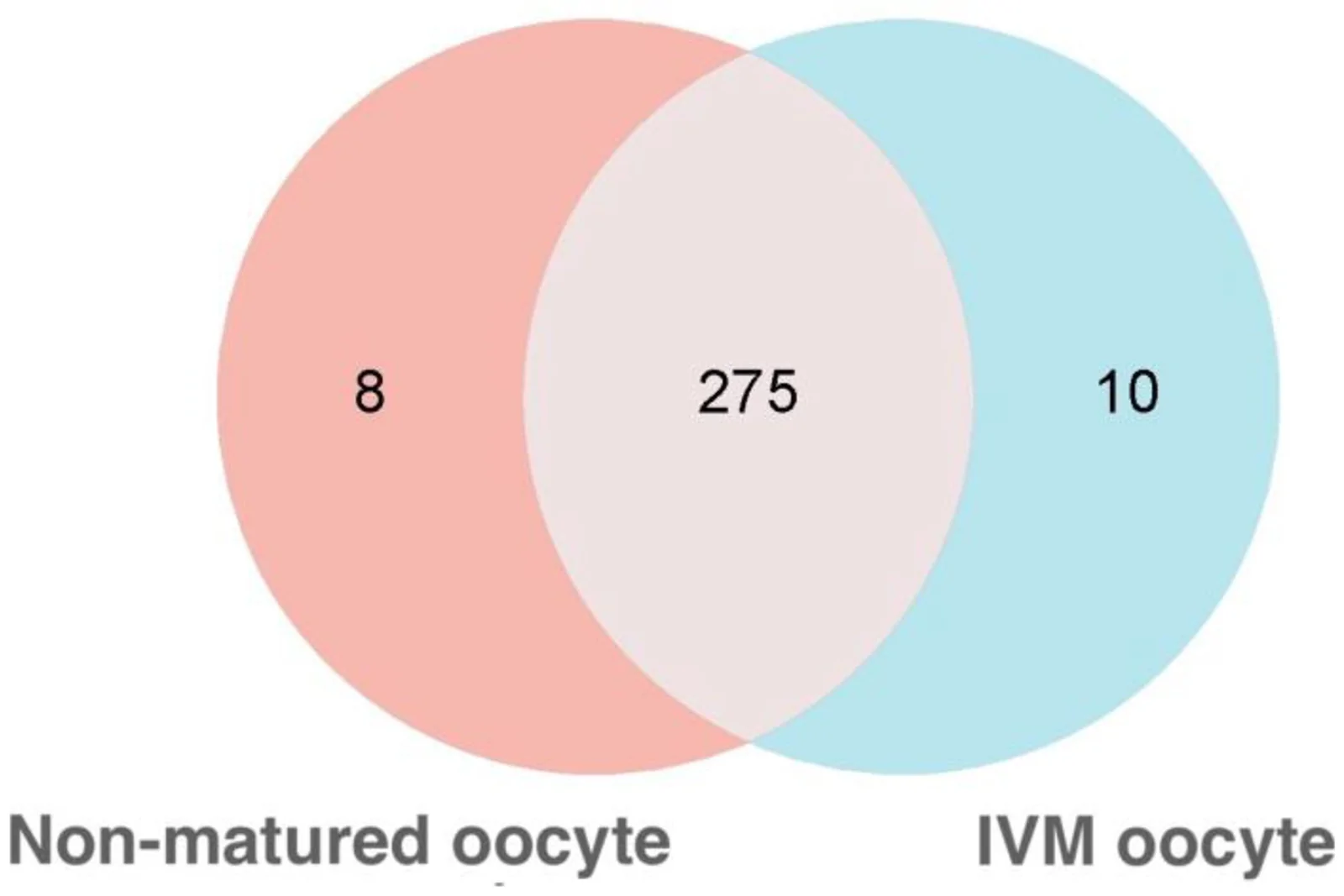
Venn diagram expressing the miRNA in non-matured and IVM oocytes.

Figure 4 shows the Venn diagram expressing the miRNA in non-matured and IVM oocytes. There were eight miRNAs expressed only in non-matured oocytes and ten unique miRNAs found exclusively after IV<M. There were 275 miRNAs shared between both conditions (Table S2, Supplementary Material).

**Figure 5:**
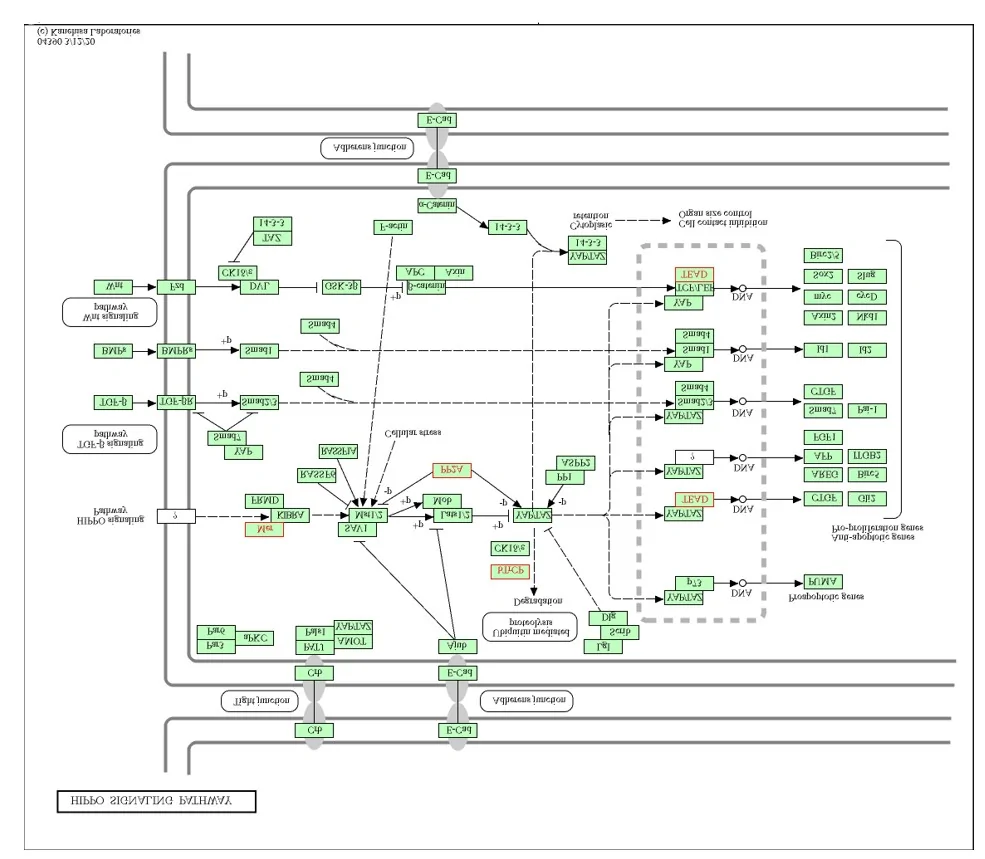
mRNA transcriptional changes represent the Hippo signaling pathway and highlight differentially expressed genes between non-matured and in vitro matured oocytes.

The transcriptional profiling of the Hippo signaling pathway in canine oocytes is shown in Figure 5, revealing a global change in gene expression following IVM. Most of the pathway components showed decreased expression, indicating a possibly widespread downregulation of Hippo signaling activity. The core regulatory kinases, such as MST1/2 and LATS1/2, exhibited reduced transcript levels. The transcripts for YAP (Yes-associated protein) and TAZ (Transcriptional co-activator) were also reduced, suggesting that the entire pathway, including its final transcriptional outputs, could be suppressed under in vitro conditions. Only a small number of genes within the pathway showed increased expression, potentially reflecting isolated compensatory mechanisms or maturation-specific responses. Genes related to pathway crosstalk, including those associated with tight junctions, gap junctions, and TGF-beta signaling, also showed signs of transcriptional repression, which might indicate a disruption in the communication between the oocyte and surrounding cumulus cells. This widespread downregulation appears to indicate suppression of Hippo signaling after IVM. A few downstream targets, such as CTGF and CYR61, showed upregulation, possibly due to a compensatory activation or selective survival differentiation signaling.

**Figure 6:**
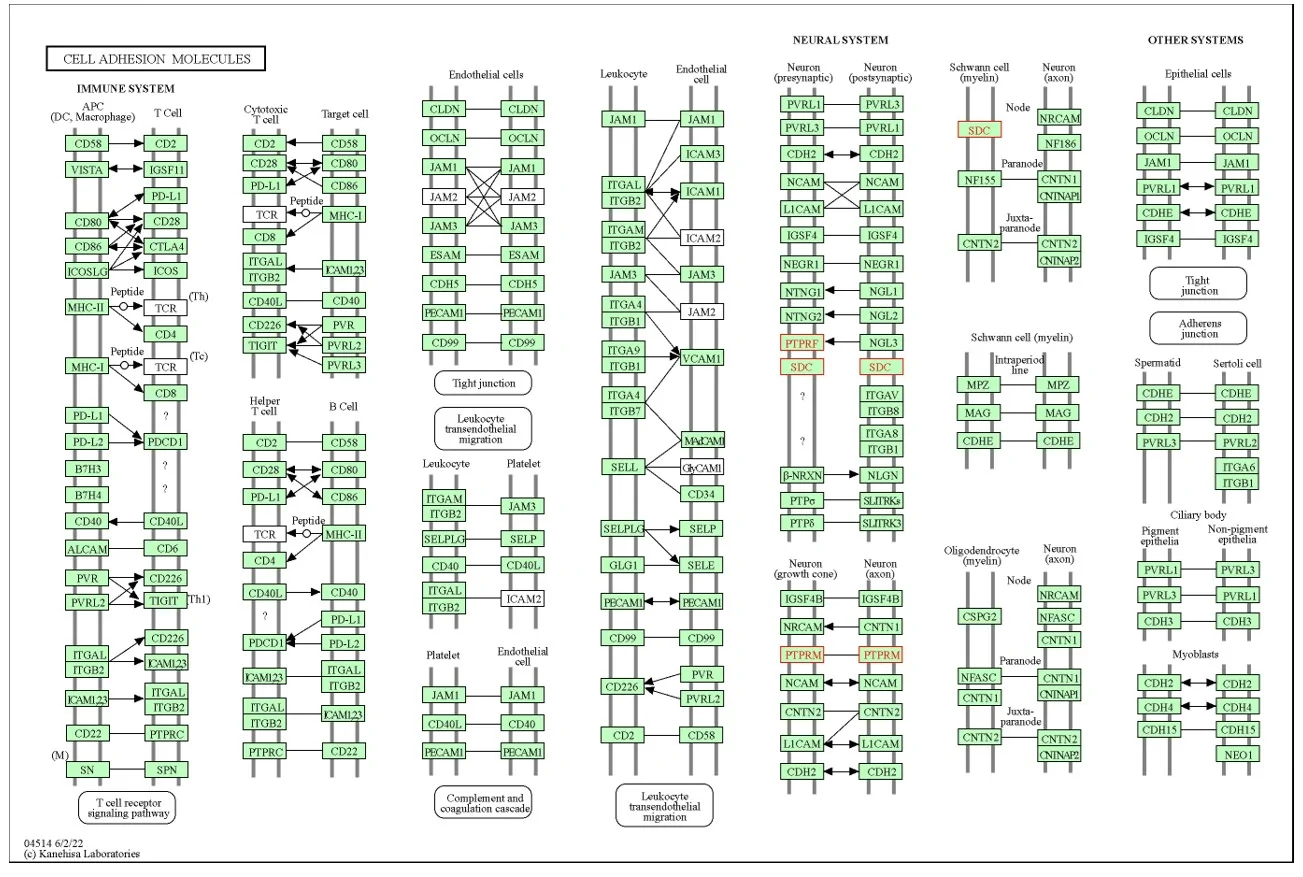
Differential mRNA expression of Cell Adhesion Molecule (CAM) signaling pathway genes during in vitro maturation of canine oocytes*.*

The Cell Adhesion Molecules (CAM) pathway is illustrated in Figure 6, organized into subfamilies that include integrins, cadherins, selectins, immunoglobulin superfamily CAMs, catenins, and other adhesion-related genes, which showed transcriptional changes when comparing non-matured and IVM canine oocytes. Most nodes, including integrin subunits such as ITGA1*,* ITGA5, and ITGB8, as well as cadherins like E-cadherin (CDH1), catenins, and IgCAM members, showed significantly reduced expression following IVM. A few upregulated genes encoding adhesion molecules were slightly increased during IVM, possibly serving as mediators of culture adaptation.

## Discussion

The unique reproductive physiology in canines presents a significant challenge for reproductive biotechnologies. Understanding the molecular dynamics is essential for identifying the barriers that limit IVM success in canines. In this study, we found significant changes in the transcriptome profiles of non-matured and IVM canine oocytes through a sequential analysis of miRNA and mRNA. At the mRNA level, a predominant pattern of downregulation was observed in non-matured oocytes, affecting key regulators of epigenetic remodeling, cytoskeletal organization, and cell signaling. At the miRNA level, differentially expressed miRNAs were identified. Pathway analysis further revealed suppression of Hippo signaling and widespread downregulation of cell adhesion molecules following IVM, suggesting a disruption in oocyte-cumulus communication and maturation-related structural remodeling. Herein, we address the biological significance of these changes in the context of canine oocyte developmental competence.

The quality of oocytes after culture was determined by morphological criteria, considering the cumulus expansion as a maturation characteristic (Nevoral et al., 2014; Ramirez et al., 2020), although we cannot ensure that all oocytes showing mucification have been in the MII stage, considering that the in vitro conditions affect the oocyte maturation process (Camargo et al., 2019). Confirmation of MII stage by chromatin visualization is incompatible with subsequent sequencing of the same pool; therefore, cumulus expansion was used as the most reliable non-destructive proxy available. Unfortunately, due to the large number of oocytes required for mRNA and especially miRNA sequencing assays, we were unable to use oocytes matured in vivo after ovulation. These limitations should be considered when interpreting our findings. While this criterion does not guarantee that all oocytes reached MII, it strongly enriches the IVM population for meiotically competent oocytes.

In the present study, the differential expression profile revealed distinct transcriptomic states between non-matured and IVM canine oocytes, where a great remodeling of the transcriptome was detected. Many RNAs accumulated in non-matured oocytes were primarily involved in transcription regulation, ribosomal or mitochondrial processes, and general housekeeping, which were downregulated or degraded. In contrast, other genes, particularly those involved in meiosis, chromosome segregation, cytoskeletal rearrangement, and cell division (Tora and Vincent, 2021; Yang et al., 2025), became upregulated. Genes that were highly expressed in non-matured oocytes but dropped after IVM might be maternal transcripts stored in the oocyte for early development, that cleared or downregulated as maturation progresses (Thelie et al., 2009). Other transcripts in IVM oocytes showed upregulation, including genes related to meiotic progression, metabolic activity, and cytoplasmic maturation (Roy et al., 2022; Xu et al., 2024), such as *ACVR1B,* which encodes a protein activin receptor-like kinase 4 (ALK-4), essential for the developmental competence of oocytes (Akimoto et al., 2023). These patterns in canines suggest that IVM triggers components of the meiotic program, although key regulators of full MII completion may not be equivalently activated under in vitro conditions. Other overexpressed genes in IVM oocytes have not been directly implicated in oocyte maturation but may have an indirect role in that function. Members of the ETS family, particularly *ETV4* and *ETV5*, play a role in enhancing oocyte maturation and ovulation in mice by upregulating *COX-2* (Eo et al., 2008; Pollard et al., 2022). In contrast, genes like *NEK7*, involved in cell cycle kinase (Gupta et al., 2017), *REPS1****,*** *BDP1* related in cell cycle control (Fairley et al., 2003), and *RANBP10* in chromatin regulation (Cao et al., 2005), showed high expression in non-matured canine oocytes, suggesting a preparatory phase, where the oocyte establishes its structural, transcriptional, and signaling characteristics (Dalbies-Tran et al., 2020; Baldini et al., 2024). In bovine oocytes, transcriptome analyses comparing immature (GV) and IVM oocytes showed thousands of transcripts enriched specifically in the GV stage, indicating that immature oocytes generally accumulate the necessary transcripts later in maturation (Mamo et al., 2011). Similarly, several maternally inherited transcripts, particularly those involved in structural and developmental programs, were highly expressed in ovine G-stage oocytes and declined as maturation progressed (Bebbere et al., 2014). In canines, a high expression of *REPS1, BDP1*, and *RANBP10,* found herein at the immature stage, is possibly involved in the molecular bases necessary for meiosis, transcriptional activation, chromatin reconfiguration, and developmental competence (Lee et al., 2021). Thus, transcripts that exhibited significant expression changes could be useful as markers to distinguish between non-mature and mature oocytes, thereby helping to assess the success of maturation, pending functional tests.

The role of miRNAs in regulating oocyte maturation by modulating the stability and translation of maternal mRNAs may be critical for meiotic progression, cytoplasmic maturation, and early embryonic development (Bartel, 2004; Assou et al., 2013). However, the miRNA-mRNA regulatory characteristics during canine oocyte maturation remain largely unexplored. Under in vitro conditions, we previously conducted a network analysis that revealed 643 significant miRNA-mRNA co-expression relationships, suggesting that miRNAs play pivotal roles in regulating mRNA expression during oocyte maturation [11]. In the present study, many miRNAs were differentially expressed between non-matured and IVM canine oocytes, suggesting a change in post-transcriptional regulation during oocyte maturation. These included those miRNAs associated with cell cycle control and developmental timing (Reza et al., 2019). In contrast, a larger group of miRNAs displayed increased expression after IVM. This implies the activation of specific miRNA-mediated gene silencing mechanisms during or because of the maturation process as described previously (Gilchrist et al., 2016), reflecting potential adaptations in signaling pathways, mitochondrial function, or apoptosis regulation, which are critical for the acquisition of developmental competence (Salilew-Wondim et al., 2020; He et al., 2021).

These trends suggest that miRNA regulation occurs during culture and indicate molecular characteristics that could support IVM in the canine model. The most important miRNAs could also be used as biomarkers for assessing oocyte maturation status.

Downregulated miRNAs in IVM oocytes: *miR-224*and *let-7g*have known roles in oocyte maturation in mice and porcine (Gong et al., 2020; Salilew-Wondim et al., 2020), folliculogenesis, and timing of meiotic resumption in other species through targeting *PTX3* in cumulus cells (Li et al., 2017). Their downregulation may reflect a loss of developmental cues during in vitro conditions or changes from growth-phase regulation.

On the other hand, the upregulated miRNAs in IVM oocytes: *miR-200c*, *miR-19b*, *miR-151*, and others, are associated with cell cycle control, apoptosis, and cell differentiation in other contexts (Baumgartner et al., 2018; Yang et al., 2025).

The overlap in miRNA expression profiles between non-matured and IVM canine oocytes showed a conserved set of miRNAs that remained active throughout the maturation process, thereby maintaining the principal post-transcriptional control mechanisms. The low number of stage-specific miRNAs observed in each group may reflect the selective remodeling of miRNA expression that accompanies the transition from the first prophase arrest. Similarly, in bovine oocytes, only a limited number of miRNAs are stage-specific (Gilchrist et al., 2016). In canids, this may be important for coordinating delayed nuclear and cytoplasmic maturation of oocytes, considering that they complete maturation post-ovulation (Reynaud et al., 2020). The miRNAs present only in non-matured oocytes may contribute to sustaining meiotic arrest and transcript stability, while those expressed only after IVM possibly promote mRNA degradation, cytoskeletal reorganization, and acquisition of developmental competence. Similar dynamic miRNA regulation has been reported in other mammals, including bovine and porcine oocytes, where specific miRNAs govern meiotic resumption, mitochondrial activation, and cumulus-oocyte signaling (Assou et al., 2013). Our findings suggest that in the canine oocyte, maturation involves not only transcriptional activation, but also fine mechanisms of post-transcriptional regulation, possibly mediated by the selective expression of miRNAs.

The Hippo signaling pathway is an evolutionarily conserved serine/threonine kinase cascade that regulates cell proliferation, polarity, cytoskeletal organization, and mechanical signaling (Avruch et al., 2012). KEGG pathway analysis herein revealed a reduction in transcript levels of several Hippo pathway components in IVM oocytes compared to non-matured oocytes. These included core kinases MST1/2 (STK3/4) and LATS1/2, as well as downstream effectors YAP1 and WWTR1 (TAZ), which regulate cytoskeletal genes and oocyte competence (Matsui and Lai, 2013; Huang and Kalderon, 2014; Clark et al., 2022). In contrast, this trend differs from that of other mammals, as active YAP1 signaling is essential for cumulus expansion and oocyte quality in bovines (Koch et al., 2022) and mice (Kawamura et al., 2013). Thus, components of the Hippo pathway remain active or increase during maturation in other species to support cytoplasmic maturation and meiotic resumption (Yu and Guan, 2013), which was not observed herein. The reduced transcript abundance of Hippo-pathway components may help explain the low in vitro maturation rates in canids.

Adhesion and cytoskeletal linkage play a central role in the maturation and developmental competence of oocytes (Santella et al., 2020). The altered expression of components involved in the cell adhesion pathway and structural integrity could also signify disruptions in the dissolution or COCs remodeling, which are critical for proper maturation (Dettleff et al., 2025). Extensive changes in adhesion-related signaling networks were observed across multiple branches in the pathway map, involving genes that mediate cell-cell contacts, extracellular matrix interactions, and cytoskeletal anchoring. The extensive regulation of adhesion and interface signaling reflects that during IVM, canine oocytes undergo remodeling of their connections to cumulus cells and extracellular matrix. In dogs, cumulus-oocyte communication remains unusually persistent (De los Reyes et al., 2020), only retracting in parallel with meiotic resumption (Dalbies-Tran et al., 2020). This suggests that the structural integrity of the COC is particularly crucial in canids and may underscore the significance of adhesion remodeling.

## Conclusion

In conclusion, this study identified distinct miRNA-mRNA regulatory networks between non-matured and IVM canine oocytes, revealing that in vitro maturation induces substantial transcriptomic reprogramming that may not fully recapitulate the molecular environment of the canine oviduct. The suppression of Hippo signaling and cell adhesion pathways, together with the differential expression of key miRNAs, suggests that oocyte-cumulus communication and epigenetic remodeling are critically compromised under current IVM conditions, likely contributing to the limited developmental competence observed in this species. These molecular characteristics provide a new framework for understanding post-transcriptional regulation during canine oocyte maturation and identify specific miRNA-mRNA networks as rational targets for optimizing IVM protocols in dogs.

## Data Availability

Data is available upon request.

## Supplementary Material

Supplementary material accompanies this paper.

Table S1 Differential gene expression data comparing mRNA between non-matured oocytes *vs*. in vitro matured (IVM) oocytes.

Table S2 miRNA expressed only in non-matured oocytes, in vitro matured (IVM) oocyte or both.

This material is available as part of the online article from https://doi.org/10.1590/1984-3143-AR2026-0003
