# Supplementary material for: Sequential miRNA-mRNA profiling of regulatory pathways during in vitro maturation of canine oocytes
Source: Anim Reprod. 2026 Aug 31;23(3):e20260003. doi: 10.1590/1984-3143-AR2026-0003 (PMC13557447; doi:10.1590/1984-3143-AR2026-0003)
Supplement: Supplementary file 1 [file 1984-3143-ar-23-3-e20260003-suppl.pdf]

Supplementary Material

Table S1: Differential gene expression data comparing mRNA between non-matured oocytes *vs.* in vitro matured (IVM) oocytes

**Accession ID**	**Gene**	**Non-matured oocyte-IVM oocyte_log2FC**	**Non-matured oocyte-IVM oocyte_adj Pval**
XM_038525746	ZNF236	-20.423556	0.00207538
XM_038526180	**REPS1**	21.2161247	0.00014501
XM_038526919	NAA35	-29.116513	4.65E-09
XM_038530448	RELL2	18.8543764	0.00296183
XM_038530541	PPP2R2B	-22.569315	2.98E-05
XM_038530796	BDP1	21.4518591	0.00015079
XM_038534761	FBXW11	-18.657024	0.01473617
XM_038534963	GRIA1	19.8850205	0.00076536
XM_038535139	PARP8	-20.978801	0.00125458
XM_038536390	ANKFY1	-20.353454	0.0020422
XM_038536729	KDM6B	-23.290219	3.40E-08
XM_038537908	TMEM201	20.6058703	0.00037668
XM_038538189	LOC119876315	21.112498	0.00022126
XM_038538520	SLC7A6	17.0157473	0.01496228
XM_038538561	RANBP10	21.039224	0.00022126
XM_038539036	AUTS2	-21.801625	0.00057617
XM_038539701	SUN1	-23.787229	2.74E-08
XM_038539844	MAZ	-30.843312	1.79E-09

XM_038541082	SORT1	21.7633827	8.75E-05
XM_038541499	DDAH1	20.1054935	0.00065736
XM_038541825	PPFIA4	-13.126721	0.02672297
XM_038541965	NEK7	24.3926155	1.21E-06
XM_038543832	PTPRM	-18.783223	0.01088801
XM_038544511	RALGAPA1	-21.199397	0.00102014
XM_038545232	MIDEAS	20.1697842	0.0005654
XM_038545383	CIPC	19.9175307	0.00076536
XM_038545404	TMED8	-24.080768	3.40E-08
XM_038546709	TTYH2	18.7641899	0.00329374
XM_038546830	BPTF	16.7962082	0.02306134
XM_038548941	SETX	20.7798721	0.00032799
XM_038549437	ZBTB6	22.2638944	3.48E-05
XM_038549447	RC3H2	21.8245688	8.71E-05
XM_038550285	MPPED1	21.3588842	0.00015079
XM_038550954	MAP4K4	19.4845282	0.00125458
XM_038551859	SEMA6A	20.5964643	0.00043746
XM_038553955	KCNK5	-18.08053	0.02306134
XM_038554554	SENP6	23.4676666	5.23E-06
XM_038554666	ZNF292	-20.468536	0.0020422
XM_038554749	MANEA	-19.365863	0.00609814
XM_038556143	PDCL2	-25.740207	3.46E-07
XM_038556635	POT1	22.2850518	4.41E-05
XM_038556969	ETV1	-20.694972	0.00168711
XM_038557925	AGO4	23.6599629	4.27E-06

XM_038558179	POMGNT1	-19.981847	0.00329374
XM_038558359	PTPRF	22.4922886	2.98E-05
XM_038558774	BLTP3B	19.8665897	0.00075158
XM_038559378	DENND11	-20.612654	0.00180326
XM_038560047	NSD3	-24.258788	2.18E-08
XM_038560712	KIDINS220	21.9563307	6.45E-05
XM_038560854	SDC1	19.533533	0.00125458
XM_038560861	PUM2	-23.636155	2.74E-08
XM_038561145	RBKS	-20.436954	0.00203202
XM_038561231	BIRC6	-21.661548	0.00072825
XM_038562203	GABPB2	-19.978959	0.00332868
XM_038564817	SAP130	19.7421283	0.00092379
XM_038565045	CCNT2	-19.404277	0.00572947
XM_038565399	ZNF35	-21.494561	0.00076536
XM_038565762	SETD5	19.9054169	0.00077667
XM_038565882	LOC119877314	-24.100187	3.03E-06
XM_038566670	FYCO1	-20.590532	0.00188183
XM_038566944	CPAMD8	12.4950942	0.00191628
XM_038567386	ZNF699	22.6823709	2.41E-05
XM_038567710	DPP9	-20.910682	0.00125458
XM_038568849	HBE1	-32.49504	1.92E10
XM_038569059	TEAD1	20.5918587	0.00037668
XM_038569113	RRAS2	-21.295137	0.00092379
XM_038570662	SLC4A7	-21.664038	0.00070264
XM_038570663	SLC4A7	20.9919534	0.00025763

XM_038571757	LZTS3	-19.065727	0.00778191
XM_038573988	RHBDD1	-21.990963	0.00050385
XM_038574083	TRIP12	-26.259246	7.46E-07
XM_038575584	NF2	-21.624229	0.00070026
XM_038576001	PPM1F	19.4372345	0.00125458
XM_038577420	SCAF11	-24.007202	3.21E-08
XM_038577506	KANSL2	-20.15033	0.00273191
XM_038577720	ACVR1B	-20.398755	0.00207538
XM_038578242	PDLIM1	-18.451512	0.01530063
XM_038578408	ARHGAP19	-20.50489	0.00203202
XM_038578547	SEMA4G	19.0415413	0.00204754
XM_038578691	NEURL1	-17.690231	0.03341401
XM_038579304	ATP6V1H	22.6921772	2.38E-05
XM_038580075	KNL1	-20.66997	0.0017207
XM_038580703	ZNF609	-19.953816	0.00307712
XM_038581279	GABPA	-19.060338	0.00918212
XM_038581724	LOC119877785	-24.969123	1.28E-06
XM_038582115	PDLIM5	20.246126	0.00065736
XM_038582603	TOMM70	20.9114897	0.00027705
XM_038582992	GOLGB1	-19.047644	0.00685535
XM_038583434	USP13	-21.631126	0.00072122
XM_038584856	RAPGEF4	21.3710892	0.00015079
XM_038585045	NCKAP1	20.4036129	0.00056396
XM_038585961	ZC3H11A	-22.146642	0.00037794
XM_038586033	CDK18	-22.137837	0.00046266

XM_038586249	DDR2	22.2887651	4.03E-05
XM_038587374	HDAC6	20.0686854	0.00070189
XM_038587624	KIF4A	21.3191355	0.00016824
XM_038587993	RNF128	-31.723979	3.94E-10
XM_038588037	LOC102157101	21.307044	0.00017964
XM_038588115	LRCH2	21.0811121	0.00022126
XR_005374239	LOC102154499	-29.884557	4.70E-09
XR_005387466	LOC119879328	-10.688524	0.04175018

Table S2: miRNA expressed only in non-matured oocytes, in vitro matured (IVM) oocyte or both.

microRNA expressed only in non-matured oocytes	microRNA expressed only in IVM oocytes	microRNA expressed in non-matured and IVM oocytes						
cfa-miR-141	cfa-miR-139	cfa-miR-148a	cfa-miR-30b	cfa-miR-708	cfa-miR-652	cfa-miR-1468	cfa-miR-184	cfa-miR-383
cfa-miR-300	cfa-miR-8903	cfa-miR-26a	cfa-miR-378	cfa-miR-497	cfa-miR-1249	cfa-miR-350	cfa-miR-205	cfa-miR-590
cfa-miR-138b	cfa-miR-216a	cfa-miR-99a	cfa-miR-152	cfa-miR-340	cfa-miR-8884	cfa-miR-1296	cfa-miR-551a	cfa-miR-6529
cfa-miR-506	cfa-miR-376b	cfa-let-7a	cfa-miR-195	cfa-miR-500	cfa-miR-194	cfa-miR-92b	cfa-miR-329b	cfa-miR-615
cfa-miR-33a	cfa-miR-376c	cfa-miR-10b	cfa-miR-486	cfa-miR-452	cfa-miR-204	cfa-miR-34c	cfa-miR-433	cfa-miR-421
cfa-miR-448	cfa-miR-8905	cfa-let-7f	cfa-miR-27a	cfa-miR-221	cfa-miR-129	cfa-miR-369	cfa-miR-32	cfa-miR-449a
cfa-miR-653	cfa-miR-1837	cfa-miR-143	cfa-miR-423a	cfa-miR-106a	cfa-miR-1343	cfa-miR-382	cfa-miR-96	cfa-miR-335
cfa-miR-8883	cfa-miR-582	cfa-miR-30d	cfa-miR-127	cfa-miR-503	cfa-miR-409	cfa-miR-8859b	cfa-miR-505	cfa-miR-664
	cfa-miR-449b	cfa-let-7g	cfa-miR-181b	cfa-miR-222	cfa-miR-328	cfa-miR-200a	cfa-miR-628	cfa-miR-188
	cfa-miR-491	cfa-miR-27b	cfa-miR-7	cfa-miR-374a	cfa-miR-1	cfa-miR-215	cfa-miR-758	cfa-miR-2114
		cfa-miR-199	cfa-miR-98	cfa-miR-224	cfa-miR-432	cfa-miR-181c	cfa-miR-20b	cfa-miR-216b
		cfa-let-7b	cfa-miR-146a	cfa-miR-363	cfa-miR-193a	cfa-miR-135a-5p	cfa-miR-223	cfa-miR-219-5p
		cfa-miR-30a	cfa-miR-339	cfa-miR-149	cfa-miR-302a	cfa-miR-299	cfa-miR-144	cfa-miR-487a
		cfa-miR-181a	cfa-miR-192	cfa-miR-196a	cfa-miR-34a	cfa-miR-193b	cfa-miR-200b	cfa-miR-8803
		cfa-let-7e	cfa-miR-532	cfa-miR-542	cfa-miR-182	cfa-miR-485	cfa-miR-153	cfa-miR-1840
		cfa-let-7c	cfa-miR-101	cfa-miR-361	cfa-miR-203	cfa-miR-889	cfa-miR-211	cfa-miR-8829
		cfa-miR-451	cfa-miR-1271	cfa-miR-1839	cfa-miR-424	cfa-miR-671	cfa-miR-190b	cfa-miR-8908b
		cfa-miR-574	cfa-miR-30e	cfa-miR-132	cfa-miR-1838	cfa-miR-486-3p	cfa-miR-345	cfa-miR-190a
		cfa-miR-21	cfa-miR-128	cfa-let-7d	cfa-miR-1306	cfa-miR-323	cfa-miR-380	cfa-miR-377
		cfa-miR-25	cfa-miR-214	cfa-miR-130a	cfa-miR-1301	cfa-miR-543	cfa-miR-1842	cfa-miR-539

		cfa-miR-26b	cfa-miR-450b	cfa-miR-95	cfa-miR-206	cfa-miR-1307	cfa-miR-346	cfa-miR-2387
		cfa-miR-30c	cfa-miR-16	cfa-miR-374b	cfa-miR-494	cfa-miR-885	cfa-miR-6516	cfa-miR-302c
		cfa-miR-125a	cfa-miR-218	cfa-miR-151	cfa-miR-126	cfa-miR-105b	cfa-miR-324	cfa-miR-483
		cfa-miR-125b	cfa-miR-8859a	cfa-miR-23a	cfa-miR-362	cfa-miR-490	cfa-miR-136	cfa-miR-8876
		cfa-miR-92a	cfa-miR-28	cfa-miR-331	cfa-miR-495	cfa-miR-217	cfa-miR-17	cfa-miR-147
		cfa-miR-191	cfa-miR-450a	cfa-miR-499	cfa-miR-8865	cfa-miR-8902	cfa-miR-454	cfa-miR-1841
		cfa-miR-10a	cfa-miR-20a	cfa-miR-146b	cfa-miR-150	cfa-miR-15a	cfa-miR-8908a-5p	cfa-miR-301a
		cfa-miR-145	cfa-miR-93	cfa-miR-105a	cfa-miR-142	cfa-miR-8908a-3p	cfa-miR-1185	cfa-miR-329a
		cfa-miR-202	cfa-miR-185	cfa-miR-181d	cfa-miR-29c	cfa-miR-487b	cfa-miR-376a	cfa-miR-34b
		cfa-miR-186	cfa-miR-122	cfa-miR-504	cfa-miR-134	cfa-miR-429	cfa-miR-1836	cfa-miR-508a
		cfa-miR-24	cfa-miR-379	cfa-miR-31	cfa-miR-200c	cfa-miR-338	cfa-miR-375	cfa-miR-551b
		cfa-miR-455	cfa-miR-411	cfa-miR-23b	cfa-miR-330	cfa-miR-676	cfa-miR-410	cfa-miR-18b
		cfa-miR-107	cfa-miR-502	cfa-miR-370	cfa-miR-19b	cfa-miR-592	cfa-miR-135a-3p	cfa-miR-2483
		cfa-miR-103	cfa-miR-155	cfa-miR-342	cfa-miR-183	cfa-miR-187	cfa-miR-138a	cfa-miR-371
		cfa-miR-148b	cfa-miR-15b	cfa-miR-425	cfa-miR-29b	cfa-miR-769	cfa-miR-18a	cfa-miR-8890
		cfa-miR-381	cfa-miR-9	cfa-miR-210	cfa-miR-212	cfa-miR-130b	cfa-miR-8908d	
		cfa-miR-320	cfa-miR-22	cfa-miR-133c	cfa-miR-135b	cfa-miR-1844	cfa-miR-124	
		cfa-miR-99b	cfa-miR-196b	cfa-miR-133a	cfa-miR-3958	cfa-miR-493	cfa-miR-1843	
		cfa-miR-29a	cfa-miR-197	cfa-miR-874	cfa-miR-660	cfa-miR-326	cfa-miR-219-3p	
		cfa-miR-140	cfa-miR-365	cfa-miR-106b	cfa-miR-19a	cfa-miR-7180	cfa-miR-133b	
